# Case of Pemphigus Terminating Fatally

**Published:** 1814-04

**Authors:** R. A. Frogley

**Affiliations:** Hounslow


THE
### Medical and Physical Journal.

### *Case of Pemphigus terminating fatally; by*

Mr. R. A. FROGLEY.

ON the morning of February 2d, 1813, I was sent for to a young man, nineteen years of age, who had kept his bed the preceding day, complaining of common febrile symptoms. On the same evening he was affected with a violent sensation of pricking and throbbing in both his insteps, extending along the anterior part of the leg nearly to the knee. This continued with great severity for five or six hours, when, at his request, his mother examined the parts, and found them red, hot, and covered with small pimples, but not swollen. The following morning I saw him again, and found the parts where the pain had previously existed to be covered with small detached vesicles. The skin was still red about their base, but there was no tumefaction. The febrile symptoms continued.

Feb. 4th.—On visiting the patient this morning, I observed different parts of his body, especially his arms, thighs, and face, to have a red hue, and a papulous feel, about which parts he complained of pain: here also there was no tumefaction. In the roof of his mouth there were several vesicles. The feverish symptoms still continued. The vesicles first formed were now filled with a yellowish serum. In the evening of the same day the parts last inflamed exhibited a vesicular appearance, and for several succeeding days the same process continued in different parts, some vesicles having arrived at maturity, while others had just emerged from the skin. They were of all sizes intermediate between that of a pea and a very large walnut.

Feb. 6th.—About this time some of them bursting, the fluid that escaped was of a nature highly irritating. When the rupture of the vesicles became general, the discharge was so copious, that a change of bed linen six times a-day was hardly sufficient to keep the patient comfortable. The fever had hitherto been of an inflammatory kind, to moderate which the common remedies were employed, such as antimonials, salines, and aperients.

Feb. 10th.—About this time, the pulse became quicker and weaker, and the tongue assumed a typhoid appearance. For this, camphor in substance, and medicines of the cordial kind were employed. The typhoid symptoms, however, gained ground, when Peruvian bark in substance, combined with the tincture, and sulphuric acid, was administered freely. The patient was also allowed a good share of wine, brandy, and porter. These cordials were varied—one day he had a pint of wine, with a bottle of porter; another day a proportionate quantity of brandy, &c. At all times his own palate regulated the quality, and the urgency of symptoms the quantity, of stimulus that was employed. The pulse for many days was about 120, and the tongue and teeth covered with a dark crust. The distress arising from the acrimonious discharge from almost the whole of the patient's body, was with great difficulty rendered tolerable. Repeated sprinklings with a mixture of lapis calaminaris and flour afforded him most comfort, and facilitated the formation of scabs. This dangerous state of symptoms continued with with little variation till the end of the month, when they began to decline; and about the 2d of March I had confident hopes of his recovery. The discharge from the sores had lessened very much in quantity, and the fever had greatly abated.

About March 20th the discharge had entirely ceased, and the patient had recovered his appetite. He was, however, miserably reduced in every feature. An universal desquamation of the cuticle took place, and the parts where vesicles existed were covered with black incrustations, from a quarter to half an inch in thickness. As these became detached, the subjacent skin was quite sound.

Soon after this period, a small tumor, which had been situated for some time previously on the upper part of the tendo achillis of the left leg, suppurated, and became an irritable ulcer. This continued to be an obstacle in the way of his recovery, for it was a source of almost constant pain and irritation, disturbing his rest by night, and destroying his appetite by day. Every variety of application, local as well as constitutional, was employed, to allay the pain and promote the healing of this ulcer. Counter-openings were tried, to give freer exit to the discharge, which, after proving useless, were allowed to heal. Opium in large doses, and other narcotics, were exhibited very freely, as these were the only means of rendering life tolerable. After lingering till the 20th of December, he died, exhausted by his sufferings.

I trust that I have here detailed the symptoms with sufficient minuteness, to illustrate the nature and progress of this disease.*

That pemphigus is sometimes an alarming and dangerous complaint, this instance abundantly testifies; and that it is a disease of comparatively rare occurrence, may be gathered

* About three years ago an instance of this disease occurred in one of the hospitals of the metropolis, which also proved fatal. Six weeks before death the eyes became affected, and were literally melted down by suppuration. The surface of the body was universally ulcerated, and covered by scabs. The mode of treatment employed was of the cordial kind, as usually recommended. Bark and wine were administered, but I was decidedly of opinion that these remedies did harm, by increasing the fever and consequent debility. In a severe case which afterwards occurred at the Northern Dispensary, I reversed the treatment with complete success. Viewing the disease in a supposed resemblance to one form of erysipelas, I bled and purged briskly, and used lotions of cold goulard water, with calamine powder, to the excoriated parts. My patient, who had been ill three weeks, recovered rapidly.—J. W.

 from from the writings of our most eminent physicians. Dr. Cullen acknowledges never to have seen an instance of it; and Dr. Willan but very few; and the majority of cases he quotes in exemplification of his own definition, are those borrowed from the practice of others. Indeed, on perusing his publication, I can find none of the cases related so fully adequate to illustrate the pathology of the disease, as the one I have now sent you. In Dr. Willan's work there is a note containing a quotation from Dr. Hodges, who, it seems, published on the Plague in 1666. In this passage Dr. Hodges describes vesicles as arising symptomatically in that disease, attended and followed by symptoms so precisely similar to those that I have detailed, that I shall take the liberty of re-copying the whole of his words. He begins "De vesiculis pestilentialibus, anglice 'Blaines.' Papulis his in vesiculam exsurgere solenne erat cum dolore lancinante et exquisito, humore intus seroso, seu ichore, coloris ut plurimum citrini, et velut straminei, circulo diversi-colori, pluries rubicundo, munitis et circumdatis. Pustulæ hæ in quâvis corporis parte emerserunt, quarum uti statio varia, ita eorum numerus omninò incertus—in quibusdam paucæ, pluries in aliis; unam sexûs sequioris vidi totam iis scatentem; quoad magnitudinem, nulla regula assignari poterat, vesiculæ enim plurimæ fabam minorem adæquabant, quandoque tamen intumuerunt grandiusculæ nuci myristicæ haud impares. Liquor inclusus suppurationis omnino incapax erat, qui utpote salinus, et fere causticus, post tantillam moram eruptâ vel corrosa cysti effundebatur, cujus color nunc citrinus, nunc lividus, nunc nigerrimus."*—*De Pest. Lond.* page 120.

*Hounslow,* R. A. FROGLEY.

*March* 5, 1814.

*For*

---

* The Editors have translated this quotation from Hodges, and have thought it right to add to it another paragraph, which seemed sufficiently important, and which is all that is written by the author on this subject.

"These vesications used commonly to rise with an exquisite and shooting pain, containing a serous humour or *ichor,* for the most part of a yellowish or straw color, and encompassed with a variegated circle, generally reddish.

"These pustules broke out in many parts of the body, and as their station was various, so their number was also uncertain. In some they were few, in others many; and a woman I once met with covered all over with them. As to their bigness, they were also uncertain; for some were as a small pea, while others increased to the magnitude of a nutmeg.

